# Identification of Aptamers that Specifically Bind to A_1_ Antigen by Performing Cell-on Human Erythrocytes

**DOI:** 10.31661/gmj.v9i0.1657

**Published:** 2020-06-27

**Authors:** Seyed Mohammad Hasan Hosseini, Mohammad Reza Bassami, Alireza Haghparast, Mojtaba Sankian, Gholamreza Hashemi Tabar

**Affiliations:** ^1^Department of Pathobiology, Faculty of Veterinary Medicine, Ferdowsi University of Mashhad, Mashhad, Iran; ^2^Institute of Biotechnology, Ferdowsi University of Mashhad, Mashhad, Iran; ^3^Immunology Research Center, Medical School, Mashhad University of Medical Sciences, Mashhad, Iran

**Keywords:** ABO Blood-Group System, Antibodies, SELEX Aptamer Technique, Flow Cytometry

## Abstract

**Background:**

The apply of aptamers as a new generation’s way to probe diagnostic for the detection of target molecules has gained ground. Aptamers can be used as alternatives to diagnostic antibodies for detection of blood groups due to their unique features. This study was aimed to produce DNA diagnostic aptamer detecting the antigen of A_1_ blood group using the Cell-Selex method.

**Materials and Methods:**

DNA aptamer was isolated against A_1_ RBC antigen after ten stages of Cell-Selex and amplification by an asymmetric polymerase chain reaction. The progress of the stages of selection was evaluated using flow cytometry analysis, which the DNA aptamer isolated from the tenth cycle with an affinity of 70% fluorescent intensity, was selected from four positive colonies followed by determination of the sequences and secondary structures.

**Results:**

The aptameric sequence obtained from C_4_ cloning was calculated with the highest binding affinity to A_1_ antigen having an apparent dissociation constant (Kd value) of at least 29.5 ± 4.3 Pmol, which was introduced as the selected aptamer-based on ΔG obtained from a colony of C_4_ equal to –13.13.

**Conclusion:**

The aptamer obtained from using Cell-Selex method could be used as an example for the development of diagnostic tools such as biosensors for detecting A_1_ blood group antigens.

## Introduction

One of the first and most basic tests in transfusion bank is to determine the grouping of antigens on red blood cell (RBC) surface based on the ABO blood group system. The results of the ABO blood group system are effective in determining the transfused blood type and conducting transplantation of solid organs. Nowadays, the ABO blood group antigens are recognized by serological methods using antibodies [1,2]. Serological methods for determining blood groups have some problems, including inconsistency of results of cell and serum grouping, technical errors, presence of alloantibodies, and other disadvantages and limitations of using antibodies (e.g., limitations of polyclonal and monoclonal antibodies, high costs, prolonged and delicate production process, difficulty in storage and transportation, and the need for specific situations) [3,4]. All this leads to the development of strategies aimed at producing an alternative structure that can be easily chosen, manufactured, and manipulated by standard biomolecular techniques [5,6]. One of the new tools are aptamers that can have a better activity than antibodies in the diagnosis, control, and drug delivery as novel biomarkers both in vitro and in vivo [6]. Aptamers are short-chain, single-stranded DNA or RNA with a length of 90–100 nucleotides. They can bind to targets including protein, peptides, amino acids, ATP, antibodies, small chemical molecules, viruses, whole cells, cellular receptors, antigens, metal ions, and enzymes based on their complex 3-D constructs. They have high specificity and binding affinity as well as dissociation constants in the range of nano- and picomolar [5]. Production, development, and replacement of aptamers with monoclonal antibodies are important for comparative functional advantages. The basic parameters of aptamers include the availability of raw materials, less complicated production methods, easy amplification and storage along with long shelf life, less technical problems and complexity, low cost, high purity, good quality, stability, and high diversity [7]. Short oligonucleotide fragments of single-stranded DNA or RNA are synthetically made in vitro using combinatory chemistry techniques. The aptamers then target nearby molecules with pre-designed specific primers by a mode of selection called systematic evolution of ligands by exponential enrichment (SELEX), and then they are amplified by polymerase chain reaction (PCR) through washing process [8]. Following amplification, the dsDNA aptamer converts into a single-stranded one by asymmetric PCR techniques or via lambda exonuclease, which is prepared for the rest of the process [9]. Selex cycles are sequentially repeated between 6 to 20 times to yield a nucleic acid ligand with the highest affinity to the target molecule, after which the process ceases, the selection and progress processes are monitored by screening tools like flow cytometry and ELISA to achieve the highest affinity [10,11]. Later, Birch *et al*. cloned the ligand to determine the sequence in a specified vector with the aim of drug delivery [9,12]. He identified aptamers of RBC surface proteins infected with aptameric malaria mosquitoes. Duan *et al*. developed a whole-bacterium SELEX technique to select aptamers that specifically bound to L. monocytogenes with high affinity [13]. Dwivedi *et al*. provide proof-of-concept that biotinylated aptamers selected by whole-cell SELEX can be used in a qPCR-based capture-detection platform for Salmonella Typhimurium [14]. Kazunori Ikebukuro *et al*. carried out pre-selection of PSA-binding aptamers by SELEX to obtain candidates for post-SELEX screening using GA and obtained five aptamers that bound to PSA [15]. However, no study was found to determine blood groups using aptamers. Given that most blood group variations are observed in blood group A and its subset A_1_ [16]. So, this study aimed to identify and locate DNA aptamers with a high specific affinity to A_1 _RBC using Cell-Selex approach and asymmetric amplification, which selected aptamers were confirmed by flow cytometry analysis followed by sequencing and prediction of their secondary structures that can replace antibodies.

## Materials and Methods

An amount of 1 × 10^6^ antigens are present on the surface of human RBCs. A_1_ RBC was obtained from the blood transfusion in sterilized bags with a refrigerated shelf life of 35 days. For each use within 24 h, 9.6 ml of blood with 0.4 ml of 20% citrate solution was poured in the falcon and then centrifuged at 1000 g for 20 minutes. After pelleting RBCs and removal of plasma and WBCs, RBCs were washed with PBS phosphate buffer salt three times (10 minutes per wash) at 1000 g. In order to prepare a stock or flow cytometry analysis, the cells were first examined for survival and activity, and then counted using a hemocytometer. Primers were designed depending on the conditions and requirements with help of the online tool Primer-BLAST (https://www.ncbi.nlm.nih.gov/tools/primer-blast/) and Gene runner Software version 6.5.50 (http://www.generunner.net). The forward primer was designed in a size of 21 nucleotides with the sequence 5'-CATCCATGGGAATTCGTCGAC-3' and a temperature of 59.13°C. The reverse primer was constructed by 19 nucleotides with the sequence 5'-CTTCCTAAGCTCGATCTCG-3' at 54.65 °C. The primers were ordered and prepared in two forms of the simple and fluorescent tag (fluorescein bound to 5' end) for flow cytometry analysis. The length of 38 nucleotides was meant for the randomized region. Accordingly, a synthetic single-stranded DNA library was designed with a length of 78 nucleotides in the two fixed (determinate) and variable (indeterminate and random) sections having HPLC-grade purity, and it was purchased from Metabion Company, Germany. The PCR reaction substances including Taq DNA polymerase, PCR 10x buffer, MgCl_2_ solution, 1500 m KCl, and 200 Mm Tris-HCl were purchased from Sina Clone Company, Iran. TEA10X buffer (1X buffer contains 40 mm tris, 2 mm acetic acid, and 1 mm EDTA) was purchased from Fermentas Company, Canada. DNA marker and loading paint 6X bromophenol blue-xylene cyanol were obtained from Geneone (Germany) and Uantis (USA), respectively. The safe stain paint (a kind of cyber green paint) and molecular grade agarose were obtained from Sina Clones, Iran. The water for PCR reaction was purified with a Millipore system (USA). The A_1_ polyclonal antibody for Counter Selex was purchased from Thermo Fisher Scientific, USA. TA Cloning vector and plasmid purification kits were purchased from INTRON (Seoul, South Korea). The gel extraction kit was obtained from Bioneer, South Korea.

###  Production of a Single-stranded Library

A synthetic library with 78-mer nucleotides was amplified by ten cycles of PCR. The 4-step thermal program included an initial denaturation (5 min, 95 °C, one cycle), the main denaturation (35 secs, 95°C), annealing (35 secs, 62 °C), and elongation (3 min, 72 °C), which were repeated in 35 cycles. A final elongation step was performed in one cycle at 72 °C for 3 minutes. The amplification process was carried out using primers F and R. The ssDNA was converted into dsDNA during the addition of asymmetric PCR (35 cycles) using primer F only as the dominant primer with a ratio of 20:1 at a final volume of 30μl. PCR products were loaded on a 2% agarose gel and stained with cyber green color (1:10.000). Electrophoresis was performed using a Multisub horizontal tank (Clear Company, UK) in 80 V at room temperature for 30 minutes. The products of PCR amplification (10 μl) were loaded on an agarose gel with 2 μl of the buffer paint and evaluated by GelDoc device. The Gene Tools software (Syngene, Syngene UK, Cambridge, UK, http://www.syngene.com/syngene-uk/) was used for image capture and analysis of DNA spots.

###  Aptamer Selection Process (Cell-Selex)

After determining cell viability, 500 μl of binding buffer (PBS containing magnesium and calcium) with 1 μl of the single-stranded library was added to the ready cell pellet and placed at 4 °C with gentle shaking (150 rpm) for 10 minutes. With the progress of enrichment stages, the incubation time was reduced from 1h to 30 minutes. To remove the raw unannealed sequence in each round of selection, the pellet was centrifuged (8000 rpm, 10 minutes) and then rinsed with washing buffer. Then, the cell pellet obtained from Selex was dissolved in DNase distilled water (100 ml) and incubated (70 °C, 10 min) for denaturation. The cells were then deposited by centrifugation (13000 rpm, 30 sec), and the supernatant containing aptamers attached to the cells were collected, and amplified by PCR. To limit the aptamers capable of attaching, Counter Selex was carried out on the masked RBC cells with A_1_ polyclonal antibody at the end of the third, fifth, and seventh cycles [16].

###  Flow Cytometry Analysis

 After three selection processes of DNA aptamer attached to the RBCs, the fluorescent intensity was measured at the 10th, 9th, 8th, 6th, and 4th cycles of the selection process (Rooyan Institute, Iran) to track enrichment of the selected library. First, the single-stranded aptamer labeled with FITC (50 P moles) was added to the binding buffer (100 ml), heated at 95 °C for 5 minutes, and placed on ice for 5 minutes. The aptamers were added to the RBC pellet from the Selex and incubated at 4 °C for 45 minutes. After incubation, the cells were washed twice and analyzed by flow cytometry.

###  Cloning, Sequencing, and Structural Analysis of Selected Aptamers

After 10 stages of the selection process and achieving the best results for fluorescent intensity in the 10th Selex cycle, the enriched aptamer DNA with a high binding affinity was selected, amplified with primers, cloned into PBluscript vector, and transferred to the genetically modified E.coli tpo10 cells. White colonies were collected on LB medium (Lurai-Bertani, with ampicillin at 37 °C) after 24 h. Then, colonies were confirmed by Colony-PCR method using aptamer-specific simple primers followed by selection of positive colonies for evaluation and characterization. Positive colonies containing aptamer sequences were amplified with primers labeled with FITC, and the fluorescence intensity was measured using flow cytometry analysis. The aptamers showing the highest binding affinity to A_1_ RBC antigen was selected for sequencing.

###  Determining the Secondary Structures of Selected Sequence 

The secondary structures of the selected aptamers were analyzed and predicted by the Mfold online software version3.5 (http://Unafold.rna.albany.edu/Q=Mfold] under conditions of Na^+^ [144 mmol/L) and Mg^2+^ (0.4 mmol/L) at 21 °C [17].

###  Determining the Dissociation Constant

For evaluation of the dissociation constant or binding of fluorescent aptamers to A_1_ RBC, different concentrations of aptamers labeled with FITC were incubated at room temperature with gentle shaking for 45 minutes. A non-linear regression curve was obtained using the software Sigma plot 12.0 and the formula Y=B_MAX_ X/[Kd^+^X], where Xs are fluorescent intensity, maximum fluorescence intensity, and concentrations of aptamers, respectively.

## Results

###  Amplification of Aptamers and Optimization of the Selection Process

**Figure 1:**
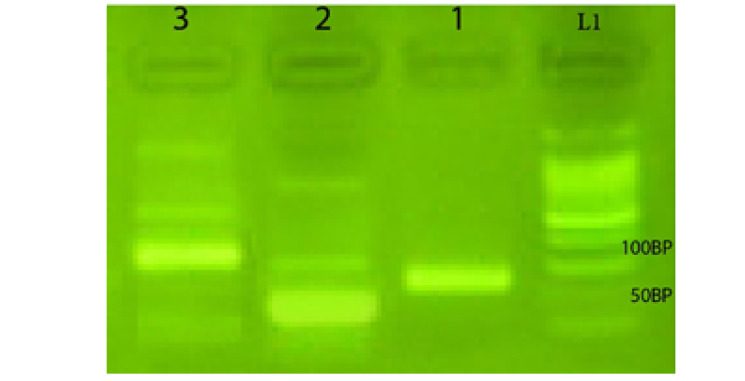
Gel electrophoresis of PCR products on agarose gel
 L1: Ladder, Row 1: PCR product, Row 2: Asymmetric PCR product by primer F
Row 3: ** Asymmetric PCR product by primer R.**

**Figure 2:**
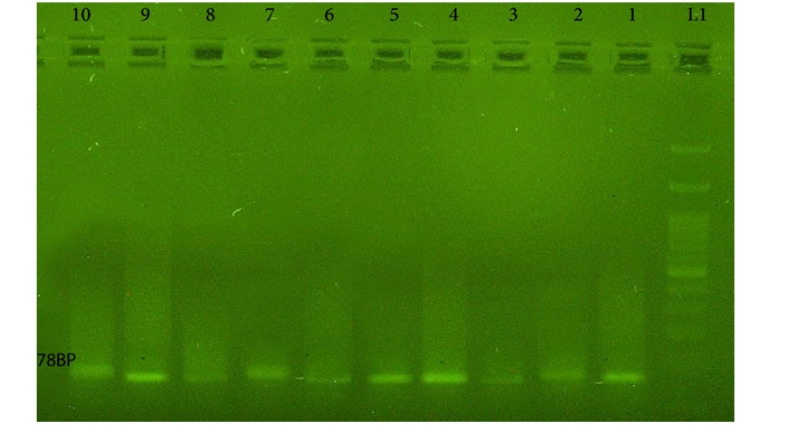
Gel electrophoresis of PCR product from the Selex selection process.

In this study, the asymmetric PCR was used to amplify a 78 nt-nucleotide library with the binding affinity to A_1_ RBC antigen. To determine the annealing temperature of PCR, four temperatures of 60, 61, 62, 63, and 64 °C were measured. Most of the amplification products formed clear bands in 78n ton gel electrophoresis at 62 °C. The primer F created a more reliable and better product. Hence, it was chosen as the dominant primer for APCR (Figure-1). A number of 30 cycles were considered for APCR amplification. Given the role of magnesium chloride in the activation of the APCR reaction and to optimize Cell-Selex method during all stages of selection, the concentration of Mg was changed from 1 mm to 0.4 mm. Electrophorus results on 2% agarose gel are exhibited in Figure-2.

###  Cell-Selex

**Figure 3:**
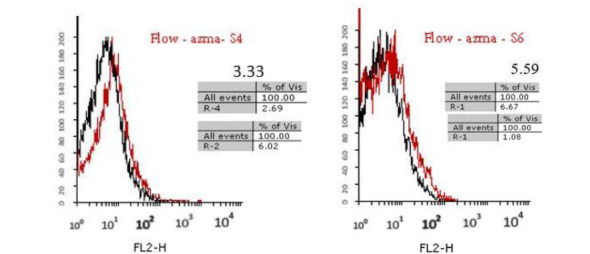
Percentage binding in Selex 4 and Selex 6.

**Figure 4:**
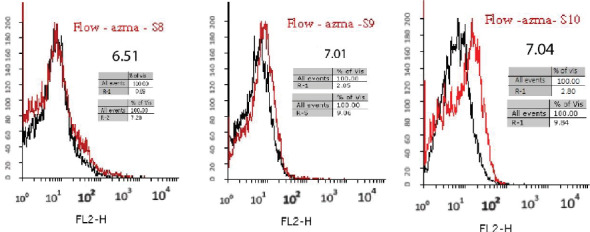
Percentage binding in Selex 8, Selex 9, and Selex 10

**Figure 5:**
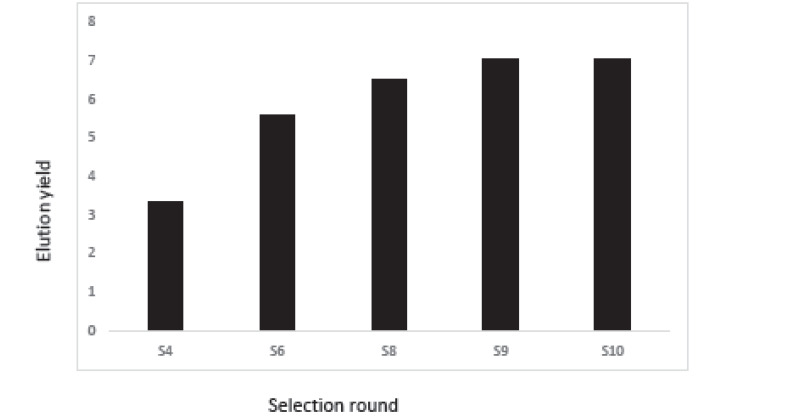
Comparing the fluorescent intensity of the selection process of Selex rounds of 4, 6, 8, 9, and 10

**Figure 6:**
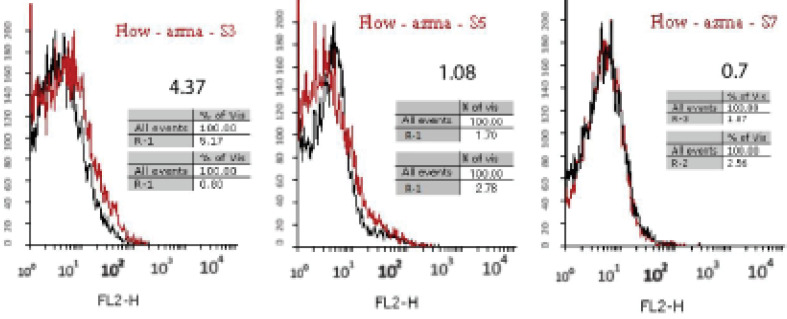
Comparing the fluorescent intensity of selection processes 3, 5, and 7 on the negative control cells

Aptamers with a specific affinity to A_1_ RBC antigen were selected following the 10th stage of the Cell-Selex process (Figure-2). Using Counter Selex, aptamers obtained from the 7th, 5th, and 3rd cycles were incubated with the RBC cells, the A_1_ antigen of which was masked with A_1_ polyclonal antibody in order to remove non-specific aptamers showing affinities to target molecules. Then, the cells were precipitated, and the supernatant containing unattached aptamers was amplified and used in subsequent stages of the selection process. The enrichment and developed aptamers with affinity to A_1_ RBC antigen were measured by fluorescence intensity of aptamers incubated in the cells at the 10th, 9th, 8th, 6th, and 4thcycles. Flow cytometry analysis yielded results of 3.3% in cycle 4 and 5.59% in cycle 6 representing a 69 percent increase in the specificity of aptamers to RBC compared to the previous steps (Figure-3). The results of the 8th, 9th, and 10th cycles were equal to 6.51%, 7.01%, and 7.04%, respectively, with increased affinity specificity of 16%, 8%, and 0.4% (Figure-4 and 5). The Selex process was deemed to be terminated with partial equality of the results of the 10th and 9th cycles. Moreover, the results of Counter Selex steps yielded results of 4.37%, 1.05%, and 0.7% for the 3rd, 5th, and 7th cycles, respectively, with the binding affinities to the masked cells equalling –35% and –75% in the 5th and 7th cycles, respectively (Figure-6).

###  Cloning and Sequencing of Enriched Aptamer

**Figure 7:**
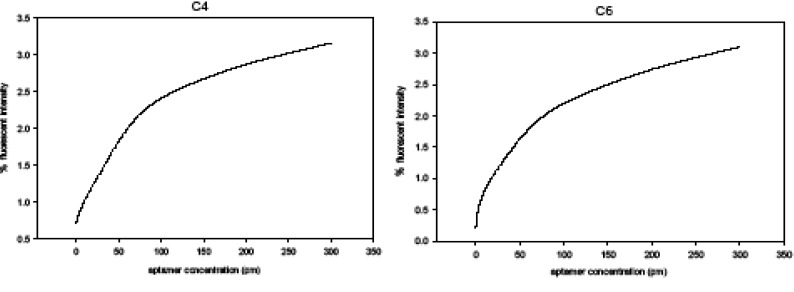
Non-linear regression curve for C4 kd clone (left) and C6 (right) to determine the binding affinity of various concentrations of aptamers C4 and C6 in relation to the cell constant.

The enriched aptamer library at the tenth stage of Selex was cloned with the highest binding affinity to A_1_ RBC. The positive colonies were selected as a result of the transformation. Of the 38 aptamers transformed, 23 clones were confirmed by the PCR-colony as positive clones. The binding intensity, analyzed by flow cytometry of four random positive colonies, namely C_2_, C_4_, C_6_, and C_8 _was equal to 0.18, 1.84, 1.01, and 0.44%, respectively. Thus, C_4_ colony with the highest intensity of fluorescence was selected and sequenced as an aptamer of choice based on the fluorescence intensity in A_1_ RBC. The effectiveness of binding affinity to the cell for the final selected aptamer compared to the clone C_6_ aptamer was assessed by flow cytometry. For this purpose, it was measured at a constant concentration of cells (2 x 10^6^) with different concentrations (0, 10, 50, 100, 200, and 300 Pmol) of aptamer labeled with FITC in a volume of 200 ml in binding buffer. The Kd values of C_4_ and C_6_ clones were assessed at 29.5 ± 4.3 Pmol/L and 32.1 ± 2.7 Pmol/L, respectively (Figure-7).

###  Determining the Secondary Structure of the Aptamer

**Figure 8:**
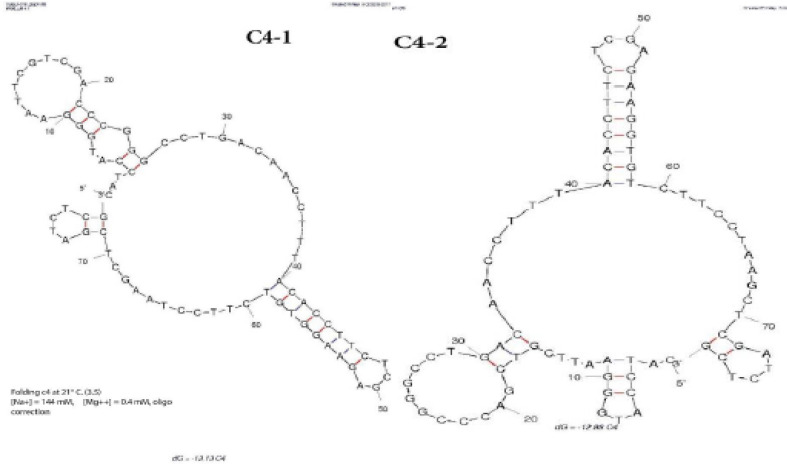
Prediction of two secondary structures for aptamer C4 based on mfold software.

The secondary structure of the DNA aptamer was calculated using the basic software, mfold, based on free energy. Minimal energy (ΔG) of –13.13 kcal/mol was calculated for the stable structure of C_4_ clone accordingly, and C_4-1_ structure was predicted as the most stable structure of the aptamer for A_1_ antigen (Figure-8). As shown in Figure 3–8, two secondary structures have been predicted for aptamer C_4_, the free energy of which are ΔG= –13.13 kcal/mol for C_4-1_ and ΔG= –12.88 kcal/mol kcal/mol for C_4-2_. According to ΔG values, the structure with the lowest ΔG value was chosen as the best structure.

## Discussion

Nowadays, aptamers are known as a new, capable, and flexible diagnostic tools as well as a convenient alternative to monoclonal antibody technology, which has been designed and produced for a lot of targets including the surfaces of human cells and pathogens [9]. Aptamers are single-strand RNA or DNA oligonucleotides that can identify a variety of target molecules by secondary structures. They are considered an important alternative due to a comparative advantage in the cost of production, ease of use, resistibility, and persistence compared to monoclonal antibodies [18]. Considering that 80 percent of people have blood group A of A_1_ type [1], this study made an attempt to design a diagnostic aptamer for A_1_ antigen using Cell-Selex method for single-stranded DNA from an oligonucleotide pool that can replace antibodies. Cell-Selex has a special advantage for creating a specific and sensitive diagnostic tool based on the detection of cell surface antigens [19]. The aptamer designed in this study was of 78-mer nucleotides in which the number of random nucleotides and the middle was 38 nucleotides. PCR was used for the amplification of double-stranded DNA resulting from each stage of the selection process. Owing to the comparative advantage of asymmetric PCR, i.e. low cost and ease of use in ssDNA production unlike other methods, the dsDNA fragments were amplified and converted into ssDNA by APCR [20]. The thermal conditions and concentration of magnesium chloride enzyme were optimized. A temperature of 62 °C was chosen for amplification. The magnesium chloride concentration of 0.4 units per ml resulted in the highest production of dsDNA. A number of 30 cycles of PCR was considered for appropriate amplification [21]. Counter Selex was used for the reduction and elimination of non-specific sequences from the masked RBCs with A_1_ polyclonal antibody during the selection process. According to the results of flow cytometry analysis of binding assays and progress of the selection process for aptamers achieved in the 4th, 6th, 8th, 9th, and 10th cycles labeled with FITC, no considerable differences were found in the 8th, 9th, and 10th cycles compared with those from the initial library. Given that there is a high difference in fluorescence intensity between the final Selex and the synthetic initial library, the selection process was deemed to be completed in 10 stages. The intensity of fluorescence in negative control cells was reduced substantially compared to the initial library. After cloning of aptamers from the 10th selection stage, four clones were analyzed by fluorescence intensity, of which the cloned C_4_ (1.84%) showed the highest affinity to A_1 _RBC antigen. The dissociation constant of the aptamer against other targets was clearly in nano-molar to pico molar ranges [14,18]. For example, the cloned aptamers to mosquito’s RBC was reported to have a dissociation constant of 14 Mm to 84 Nm [22]. The results of binding examinations in this study revealed a C_4_ equilibrium dissociation constant of 29.5±4.3 Pmol/L compared to the C_6_. Hence, C_4_ aptamer was selected as the final one. The steam and loop structures, as well as a low ΔG, are the key to the binding domain and the binding site in secondary structures of aptamer [18,23]. Of the structures obtained, C_4-1 _aptamer contains G4 and guanine-rich motifs. G4 motif can help a stable Scaffold aptamer. Of the secondary structures obtained for C_4 _aptamer, two structures were attained by the web-based mfold software via a minimum energy level at 21°C and a sodium concentration of 114 Mm. The structure of C_4-1_ (ΔG= –13. 13) was proposed as the best final structure. A specific aptamer was isolated for ARBC antigen in this study. The aptamer can detect A_1_ RBC antigen in the ABO blood group system. Ni *et al*. used white blood cells for Counter Selex [24]. White blood cells, antigen B, and other blood group antigens can be used as Counter Selex to ensure the removal of non-specific targets and non-specific aptamer binding.

## Conclusion

The aptamer designed has many advantages over A_1_ monoclonal antibody in the detection of blood group system that can replace antibodies in immunological diagnostic tests. It can also be used as part of a differential diagnostic tool such as biosensors and ELISA for the detection of the A_1_ blood group.

## Acknowledgment

 The authors are grateful to Ms. Ahadi of Rooyan Institute for provided flow cytometry analysis, and also to Dr. Kalantari, (Gholhak Laboratory) and Dr. Ali Kazemi. This study was a part of a project by Dr. Hashemitabar of the Ferdowsi University of Mashhad.

## Conflict of Interest

 The authors declare that there is no conflict of interest.

L1: **Ladder, and** rows 1 to 10: ** PCR products’ selection process of one to ten cycles.**
